# *-In silico *functional characterization of a double histone fold domain from the *Heliothis zea *virus 1

**DOI:** 10.1186/1471-2105-6-S4-S15

**Published:** 2005-12-01

**Authors:** Claudio Greco, Piercarlo Fantucci, Luca De Gioia

**Affiliations:** 1 Dipartimento di Biotecnologie e Bioscienze, Università degli Studi Milano-Bicocca, P.zza della Scienza 2, 20126 Milano, Italy

## Abstract

**Background:**

Histones are short proteins involved in chromatin packaging; in eukaryotes, two H2a-H2b and H3-H4 histone dimers form the nucleosomal core, which acts as the fundamental DNA-packaging element. The double histone fold is a rare globular protein fold in which two consecutive regions characterized by the typical structure of histones assemble together, thus originating a histone pseudodimer. This fold is included in a few prokaryotic histones and in the regulatory region of guanine nucleotide exchange factors of the Sos family. For the prokaryotic histones, there is no direct structural counterpart in the nucleosomal core particle, while the pseudodimer from Sos proteins is very similar to the dimer formed by histones H2a and H2b

**Results:**

The absence of a H3-H4-like histone pseudodimer in the available structural databases prompted us to search for proteins that could assume such fold. The application of several secondary structure prediction and fold recognition methods allowed to show that the viral protein gi|22788712 is compatible with the structure of a H3-H4-like histone pseudodimer. Further *in silico *analyses revealed that this protein module could retain the ability of mediating protein-DNA interactions, and could consequently act as a DNA-binding domain.

**Conclusion:**

Our results suggest a possible functional role in viral pathogenicity for this novel double histone fold domain; thus, the computational analyses here reported will be helpful in directing future biochemical studies on gi|22788712 protein.

## Background

DNA packaging in the nucleus of eukaryotic cells is allowed by the assembly of nucleosomal elements, which are composed by a proteic core particle around which DNA is wrapped. The nucleosomal core comprises eight histones, short basic proteins characterized by a high content of lysine and arginine. Several crystallographic and biochemical studies [1-3] have shown that histone H2a is able to form a stable complex with histone H2b, while the H3 monomer can interact with histone H4. The 3D-structure of histones is characterized by the presence of two or three short alpha-helices flanking a longer helix; each of these helices is typically amphiphilic, and the strong interaction between monomers composing a histone dimer is based on the tight packaging of their hydrophobic surfaces.

The histone fold is not a feature specific for eukaryotic histones only; in fact, this fold is also observed in a group of prokaryotic histones [4], in some transcription factors [5], and in the amino-terminal domain of the guanine nucleotide exchange factors of the Sos family [6]. Moreover, the crystallographic analysis of the human homologue of Sos1 ([7], PDB code 1q9c) and of the prokaryotic histone from *Methanopyrus kandleri *([8], PDB code 1f1e) showed the presence of two different interacting histone fold motifs localized along the same polypeptidic chain. Such a structural arrangement is referred to as "histone pseudodimer" or "double histone fold".

The amino-terminal double histone fold domain of Sos proteins is structurally very similar to the H2a-H2b histone dimer [7], while for the prokaryotic histone pseudodimer it is not possible to individuate a direct structural counterpart in the eukaryotic nucleosome core particle. Consequently, no H3-H4-like histone pseudodimer has been characterized so far.

Prompted by the above observation, we have searched for new sequences potentially compatible with the structure of a putative H3-H4 histone pseudodimer. The results from this search indicated a viral protein from the *Heliothis zea *virus 1 (Hzv-1) as a possible H3-H4 double histone fold containing protein; this structural assignment was validated by using several secondary structure prediction and fold recognition methods. Finally, the *in silico *functional characterization of this histone pseudodimer is reported.

## Methods

The initial sequence homology searches were carried out by means of transitive PSI-BLAST analyses [9]. Sequence alignments were obtained with ClustalW [10] and manually refined.

Secondary structure predictions were obtained using three different tools: PSI-Pred [11], J-pred [12] and PHD [13]. Meta-predictions were carried out by comparing the results obtained from these three servers, and taking into consideration only the sequence regions that were predicted to assume a particular secondary structure by at least two servers, with a degree of reliability of 50% or higher.

Fold recognition results were obtained using the 3D-jury meta server [14]. The servers used by 3D-jury for consensus building were: 3D-PSSM [15], Meta-Basic [16], FFAS03 [17], FUGUE2 [18], INUB [19], and mGenTHREADER [20].

The Swiss-model server [21] was used to obtain a 3D-model of the viral histone pseudodimer. The H3-H4 histone dimer from *Gallus gallus *(PDB code: 1eqz) was chosen as a template. The server generated the model in a fully automatized way, and the reliability of the result from such procedure was checked by means of PROCHEK [22]. The analysis of the model was carried out with Pymol [23] and Swiss PDB viewer [24]. Swiss PDB-viewer was also used in order to obtain the electrostatic potential map of the histone pseudodimer 3D-model.

The prediction of DNA-binding sites on the H3-H4 histone pseudodimer model was carried with the Pre-Ds server [25].

## Results and discussion

### The viral protein gi|22788712 is compatible with a H3-H4-like double histone fold

The absence of known H3-H4-like histone pseudodimers in the available structural databases did not allow to apply a standard PSI-Blast search as a starting point of the present work. Consequently, we applied a specific search strategy based on the submission to Psi-Blast of some "chimeric" sequences obtained linking different protein regions included in the H3 and H4 monomers of the histone dimer from *Gallus gallus*. In particular, the submission of a query sequence comprising the sequence segments 20–103 and 40–136 from histones H4 and H3 evidenced the existence of a viral protein (NCBI code gi|22788712) from the *Heliothis zea *virus 1 which encompasses two consecutive regions, respectively homologous to histones H4 and H3. This protein appeared already at the first iteration, and the corresponding E-value (6e-7) underlines the statistical relevance of the match. The gi|22788712 protein includes a long N-terminal module of unknown function, while the regions of homology to histone H4 (residues 905–980) and H3 (residues 990–1095) are localized along the C-terminal part of the aminoacidic sequence. Such viral polypeptide is defined as "histone H3, H4" in the corresponding NCBI record; however, this generic annotation is not sufficient to assign a double histone fold domain to this module. Actually, the formation of a histone pseudodimer is expected to require a strict conservation of hydrophobic patterns and secondary structure elements on both the histone folds [26]; moreover, the linker region between the two histone folds must be sufficiently long and flexible to allow the assumption of a globular fold. Consequently, we decided to carry out an *in silico *analysis in order to verify if this viral protein sequence is compatible with the presence of a histone pseudodimer. The computational results we obtained have been also used to propose a functional role for this protein module: in fact, viral proteins comprising histone folds are very rare, and no experimental data on them are available at present.

**Figure 1:**
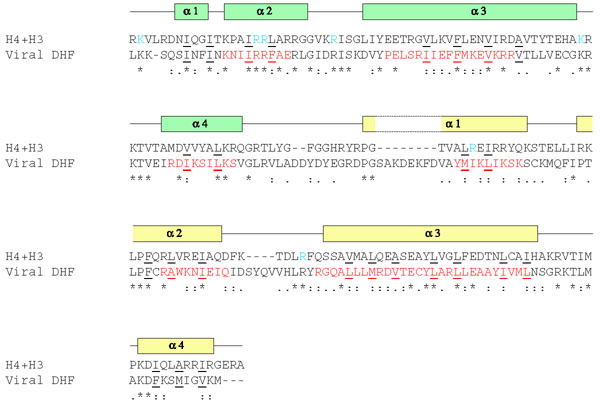
sequence alignment between "chimeric" H4-H3 histone and the double histone fold (DHF) from *Heliothis zea *virus 1. Above the alignment, are shown the positions of alpha-helices in histone H4 (green boxes) and histone H3 (yellow boxes). Red letters indicate regions predicted to assume an alpha-helical structure, based on a comparison among the results obtained from three different secondary structure prediction servers (PHD, J-pred, Psi-pred; see Methods). Light blue letters correspond to the basic residues which mediate the contact between the H3-H4 histone dimer and DNA. Underlined residues belong to the pattern of aminoacids whose hydrophobicity is strictly conserved.

The sequence alignment between nucleosomal H4 and H3 histones and the C-terminal portion of the viral protein is shown in figure 1. The percentage of identical residues shared by histones H4, H3 and the target sequence is 32,6% and 19,8% respectively. Notably, analysis of the alignment highlights a strict conservation of the hydrophobic residues involved in definition of the amphiphilic character of the alpha-helices, which is crucial for the correct folding of double histone fold domains.

An analysis based on three different secondary structure prediction servers (PHD, Jpred and Psi-PRED, see methods) was then carried out: the results obtained confirmed the structural conservation of the putative alpha-helices corresponding to those normally included in H3 and H4 histone folds (see figure 1). Moreover, all prediction servers indicated that the linker between the two histone folds in the viral protein is characterized by neither an alpha-helix nor a beta-strand conformation, thus suggesting an extended, random coil conformation for this region; this result was expected because, as mentioned above, in a histone pseudodimer the presence of a flexible spacer is necessary to allow the establishment of intramolecular interactions between the two histone folds.

In order to further validate the hypothesis that the two consecutive H3 and H4 histone folds can pack against each other giving rise to a histone pseudodimer, we submitted the corresponding sequence region from the viral protein to the fold recognition meta-server 3D-jury (see Methods). This meta-predictor indicated the structure of the double histone fold domain from *Methanopyrus kandleri *as the most suitable to describe the fold of the query sequence. Previous literature data [27] have shown that 3D-jury scores above 50 correspond to correct structure assignment in over 90% of the cases; as for the viral protein *gi*|*22788712*, the score reported by the algorithm was 68.67, well above the threshold that indicates a highly reliable structural assignment.

### *In silico *functional characterization of the viral histone pseudodimer

Double histone fold domains from *Methanopyrus kandleri *and from Sos proteins have very different biological roles: in fact, the prokaryotic histone pseudodimer is implicated in chromatin packaging [28], while Sos double histone fold domain is known to exert an inhibitory action towards the Ras-GEF activity expressed by this protein class [29]; moreover, the cytoplasmic localization of Sos proteins [7] indicate that they should not exhibit function of DNA-binding factors.

The above observations prompted us to carry out an *in silico *analysis on the novel double histone fold domain from Hzv-1, in order to suggest a possible biological role for this protein module.

**Figure 2:**
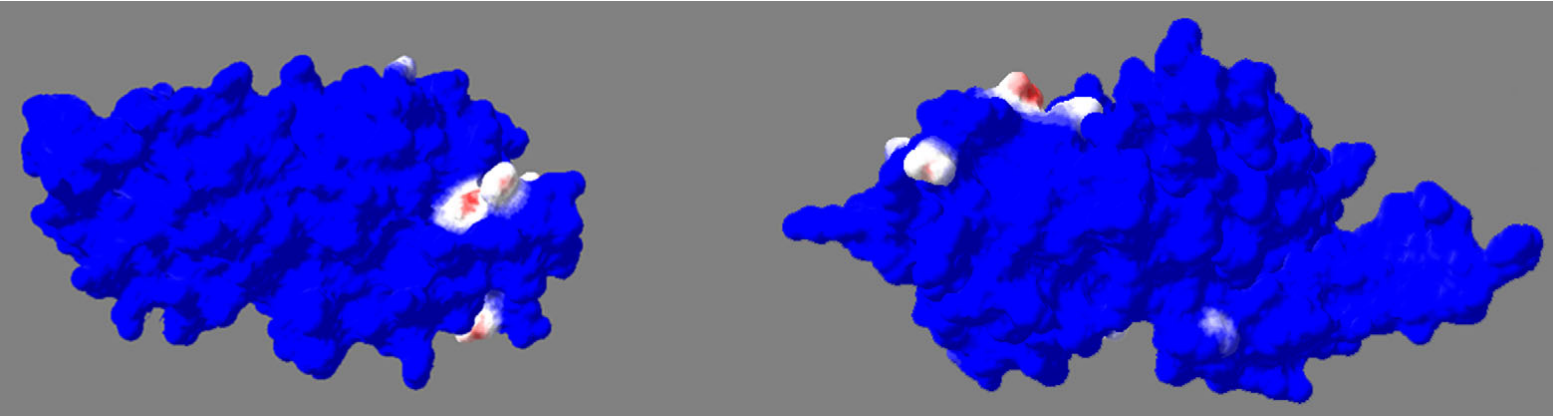
left side: molecular electrostatic potential of the viral double histone fold, in the region of putative contact between the protein and DNA. Blue surfaces correspond to repulsive regions (i.e. positively charged), while red surfaces to attractive regions (i.e. negatively charged). Right side: molecular electrostatic potential of the nucleosomal histone dimer H3-H4, in the region of contact between the dimer and DNA.

**Figure 3:**
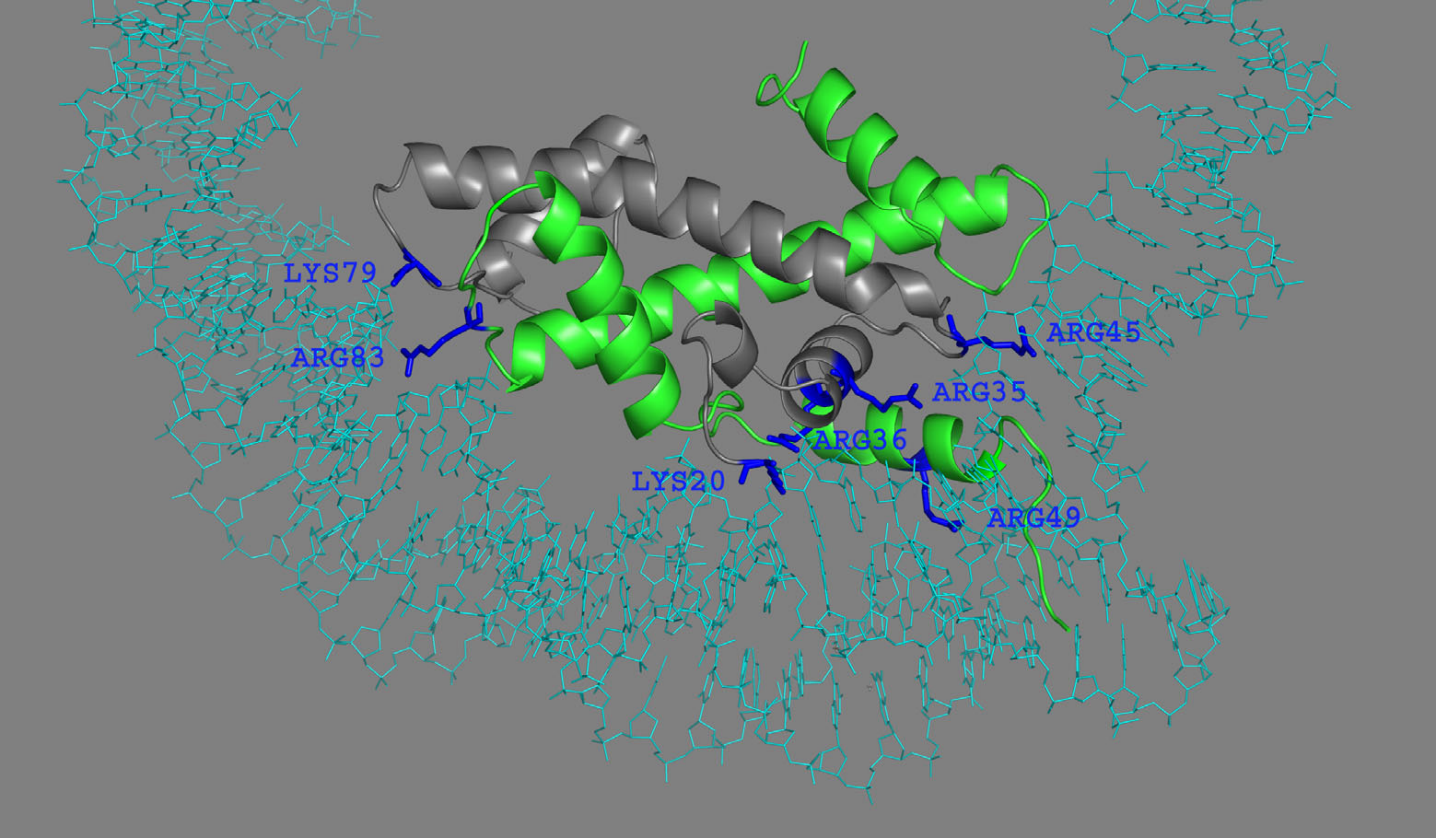
relative positions of histone H3 (in green), histone H4 (in grey) and DNA in the nucleosome core particle; the basic aminoacidic residues in direct contact with DNA are labelled and coloured in blue.

As a first step, a homology model was built for the viral histone pseudodimer (see methods); the structural reliability of the model was checked by using PROCHECK program suite [22]. The calculation of PROC-AVE parameter, (which represent a carefully weighted average of all the analyses performed by PROCHECK) gave a value of 0.13, significantly higher then the threshold of -0.5 which discriminates between poor and good models. Then, we compared the chemical-physical properties of the H3-H4 histone dimer with those of the histone pseudodimer model. In the H3-H4 nucleosomal histone dimer, the surface region that mediates protein-DNA contacts is dominated by contributions coming from basic (protonated) aminoacids; as a result, attractive interactions between the histone dimer and deoxyribonucleic acids can take place. The corresponding surface region of the viral histone pseudodimer resulted to be positively charged too, as evidenced in the electrostatic potential map shown in Figure 2; moreover, the sequence comparison between histones and the viral double histone fold evidenced that the basic residues directly involved in protein-DNA contacts (R83, R49 in histone H3, and R45, R35, R36, K20, K79 in histone H4) are generally conserved or substituted with other aminoacids that could be involved in DNA binding (Figure 1 and 3).

**Figure 4:**
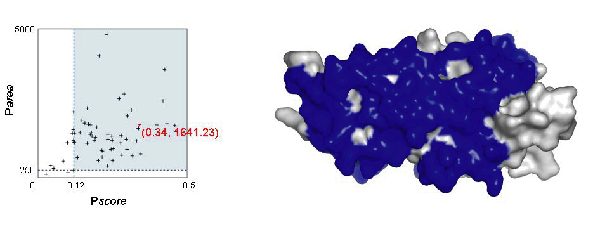
left side: graphical representation of the statistical parameters (Pscore and Parea, see [30]) on which the prediction of DNA-binding site is based. Black crosses indicate the Pscore and Parea values calculated for 63 representative dsDNA-binding proteins, while red asterisk refers to the values of the same parameters for the viral histone pseudodimer. Only proteins with Pscore > 0.12 and Parea > 250 (thus included in the upper right region of the graph) are considered dsDNA-binding proteins. Right side: localization of the predicted DNA-binding surface (in blue) on the viral histone pseudodimer model.

The availability of a model for the viral double histone fold allowed us to apply a novel and highly reliable computational method for the identification of DNA-binding proteins; this method, developed by Tsukiya et al. [30], focuses on the shape of the molecular surface of the protein and DNA and on the electrostatic potential on the surface; the resulting prediction scheme shows 86% and 96% accuracy for DNA-binding and non-DNA-binding proteins, respectively [30]. The results obtained from the application of such method were consistent with all the observations above reported: the viral histone pseudodimer was recognized as a DNA-binding module (Figure 4), and the surface portion indicated by the algorithm as the DNA-binding region on the histone pseudodimer model lies over the conserved basic surface previously described.

It is known that some DNA-virus genomes are complexed with cellular histones to form a chromatin-like structure inside the virus particle [31]. In view of this observation, and considering the results of the computational study here reported, we hypothesize that the double histone fold domain from Hzv1 could contribute to the packaging and organization of viral DNA in the capsid; however, sequence analysis of the viral histone pseudodimer also suggests a possible direct involvement of this protein domain in viral pathogenicity. In fact, the amino-terminal tails of histones H3 and H4 have a fundamental role in the modulation of histones-DNA interaction; consequently, mutations and deletion in these regions can determine a negative effect on nuclear DNA replication and cell cycle progression [32,33]; notably, these regions are the less conserved in the viral double histone fold sequence, and the expression of such a DNA binding domain in cells infected by the Hzv-1 could interfere with physiological processes of crucial importance for cell growth. However, on such basis our hypothesis would remain speculative, and future biochemical studies will thus be required for its validation.

## Conclusion

The double histone fold is an all-alpha protein fold characterized by the tight interaction between two distinct histone folds belonging to the same peptide chain. Previously, this fold has been recognized only in the guanine nucleotide exchange factors of the Sos family and in a few prokaryotic histones.

Sequence analyses, coupled with results from several secondary structure prediction and fold recognition algorithms, allowed to show that also the viral protein gi|22788712 can be included in the group of proteins containing a double histone fold. Further structure-function relationship studies revealed that the chemical-physical properties of the viral histone pseudodimer are compatible with DNA binding; our *in silico *results will be helpful in directing targeted biochemical studies aiming at the experimental functional characterization of this interesting viral protein domain.

## Authors' contributions

C.G. conceived the idea, carried out the sequence and structure analysis and drafted the manuscript. P.F. provided general guidance in the project. L.D.G. participated in the design of the study and prepared the final version of the paper. All authors read and approved the final manuscript.
